# Research hotspots and future trends in lipid metabolism in chronic kidney disease: a bibliometric and visualization analysis from 2004 to 2023

**DOI:** 10.3389/fphar.2024.1401939

**Published:** 2024-09-03

**Authors:** Ying Wang, Tongtong Liu, Weijing Liu, Hailing Zhao, Ping Li

**Affiliations:** ^1^ Dongzhimen Hospital, Beijing University of Chinese Medicine, Beijing, China; ^2^ Guang’anmen Hospital, China Academy of Chinese Medical Sciences, Beijing, China; ^3^ China-Japan Friendship Hospital, Institute of Medical Science, Beijing, China

**Keywords:** bibliometrics, chronic kidney disease, lipid metabolism, visualization, citespace

## Abstract

**Background:**

Disorders of lipid metabolism play a key role in the initiation and progression of chronic kidney disease (CKD). Recently, research on lipid metabolism in CKD has rapidly increased worldwide. However, comprehensive bibliometric analyses in this field are lacking. Therefore, this study aimed to evaluate publications in the field of lipid metabolism in CKD over the past 20 years based on bibliometric analysis methods to understand the important achievements, popular research topics, and emerging thematic trends.

**Methods:**

Literature on lipid metabolism in CKD, published between 2004 and 2023, was retrieved from the Web of Science Core Collection. The VOSviewer (v.1.6.19), CiteSpace (v.6.3 R1), R language (v.4.3.2), and Bibliometrix (v.4.1.4) packages (https://www.bibliometrix.org) were used for the bibliometric analysis and visualization. Annual output, author, country, institution, journal, cited literature, co-cited literature, and keywords were also included. The citation frequency and H-index were used to evaluate quality and influence.

**Results:**

In total, 1,285 publications in the field of lipid metabolism in CKD were identified in this study. A total of 7,615 authors from 1,885 institutions in 69 countries and regions published articles in 466 journals. Among them, China was the most productive (368 articles), and the United States had the most citations (17,880 times) and the highest H-index (75). Vaziri Nosratola D, Levi Moshe, Fornoni Alessia, Zhao Yingyong, and Merscher Sandra emerged as core authors. Levi Moshe (2,247 times) and Vaziri Nosratola D (1,969 times) were also authors of the top two most cited publications. The International Journal of Molecular Sciences and Kidney International are the most published and cited journals in this field, respectively. Cardiovascular disease (CVD) and diabetic kidney disease (DKD) have attracted significant attention in the field of lipid metabolism. Oxidative stress, inflammation, insulin resistance, autophagy, and cell death are the key research topics in this field.

**Conclusion:**

Through bibliometric analysis, the current status and global trends in lipid metabolism in CKD were demonstrated. CVD and DKD are closely associated with the lipid metabolism of patients with CKD. Future studies should focus on effective CKD treatments using lipid-lowering targets.

## 1 Introduction

Chronic kidney disease (CKD) is an incurable progressive disease with high morbidity and mortality (Kalantar-Zadeh et al., 2021). It is an increasingly underappreciated global public health problem that imposes huge economic and medical burdens on society and health institutions (Eckardt et al., 2013). The current global prevalence of CKD is estimated to range from approximately 11%–13% (Hill et al., 2016). By contrast, the absolute risk of death increased exponentially with a decline in renal function (Tonelli et al., 2006). Patients with CKD are five to ten times more likely to die prematurely than those who progress to end-stage renal disease (ESRD) (Webster et al., 2017). Slowing down the progression of CKD is a global medical priority. Presently, dietary intervention and drug therapy for CKD can maximize the preservation of renal function. However, patients with CKD progress to ESRD, and renal replacement therapy is inevitable (Kalantar-Zadeh et al., 2021). CKD is heterogeneous in terms of risk, etiology, and clinical presentation. It leads to abnormal lipid metabolism and changes in lipid levels, composition, and quality (Ferro et al., 2019), and lipid accumulation in the renal parenchyma may lead to inflammation and fibrosis (Mitrofanova et al., 2023). Dyslipidemia in CKD is primarily caused by increased triglyceride (TG) levels, decreased high-density lipoprotein cholesterol (HDL-C) levels, and changes in low-density lipoprotein cholesterol levels (Hager et al., 2017). Very low-density lipoprotein, HDL, lipid concentration, and the composition of these lipoproteins, as well as TG and fatty acids in all lipoprotein subclasses, are associated with CKD risk (Geng et al., 2024). Thus, lipid metabolism is a crucial area of CKD research.

Publications are representative of the results of scientific research. Bibliometric analysis can be used to analyze the development trend and core influence of a specific scientific field (Chen and Song, 2019). Bibliometrics is mainly analyzed in terms of performance and visualization (Nakagawa et al., 2019; Sabe et al., 2023). There are currently several publications in the fields of CKD and lipid metabolism. However, there have been no systematic bibliometric evaluations of lipid metabolism in patients with CKD. Therefore, we conducted a bibliometric study to comprehensively evaluate the topic hotspots and trends in CKD and lipid metabolism.

## 2 Methods

### 2.1 Bibliometric data and search strategy

**FIGURE 1:**
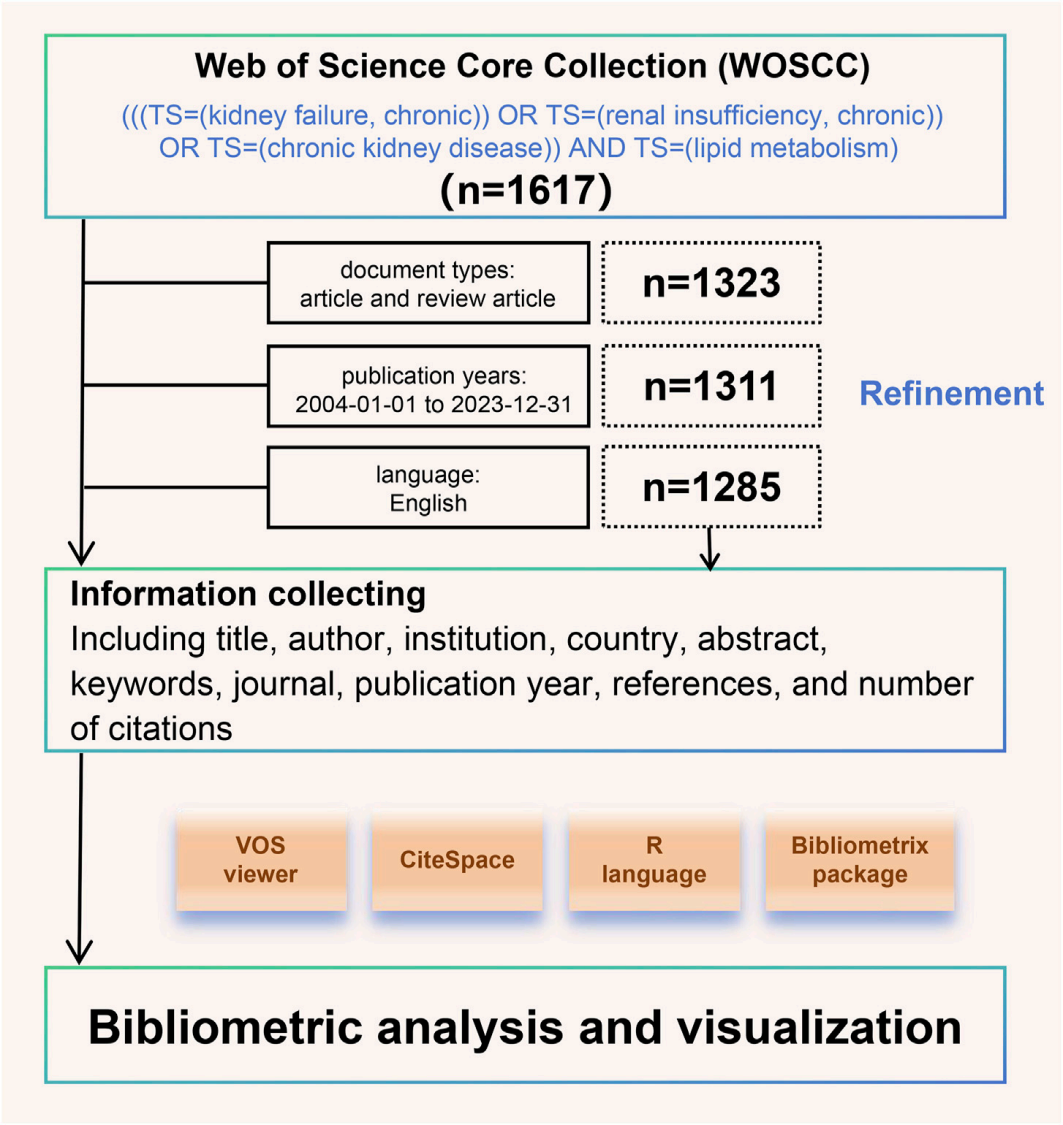
Publications refinement and bibliometrics analysis process.

Since the inception of the database, we have searched the Science Citation Index Expanded and Social Sciences Citation Index of the Web of Science Core Collection (WOSCC) for literature on CKD and lipid metabolism. The search formula was as follows: (((TS = (kidney failure, chronic)) OR TS = (renal insufficiency, chronic)) OR TS = (chronic kidney disease)) AND TS = (lipid metabolism). Simultaneously, we excluded studies based on publication date, literature type, and language, as detailed in Figure 1.

### 2.2 Data analysis and bibliometric analysis

We collected information on all eligible articles, including title, author, institution, country, abstract, keywords, journal, publication year, references, and number of citations. All of these were stored in a downloadable format. This study used VOSviewer (v.1.6.19), CiteSpace (v.6.3 R1), the R language (v.4.3.2), and the Bibliometrix (v.4.1.4) package (https://www.bibliometrix.org) for bibliometric analysis and visualization, as detailed in Figure 1. The Hirsch index (H-index) can be used to evaluate the amount and level of academic output of researchers, meaning that H of N papers published are cited at least h times (Hirsch, 2005). Burst analysis can reveal sudden changes in citations within a specific period, thereby identifying important nodes and understanding emerging research trends (Sabe et al., 2023).

## 3 Results

### 3.1 Global research status of CKD and lipid metabolism

**FIGURE 2:**
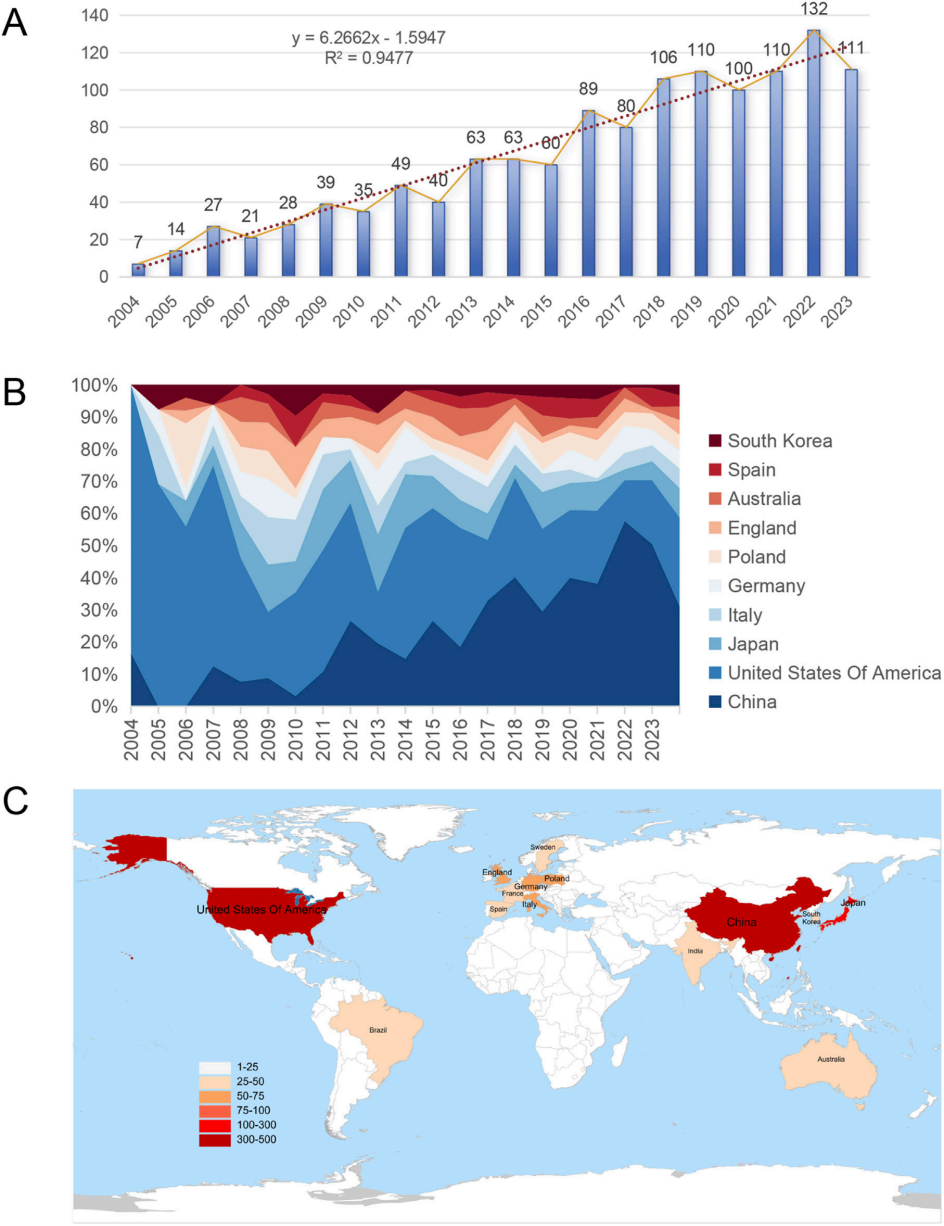
Overall global trends in relevant publications from 2004 to 2023. **(A)** Annual changes in the number of publications; **(B)** Percentage area stacking plot of the number of publications in the top ten countries over time; **(C)** Distribution of countries with more than 25 publications.

According to the above search strategy, from 2004 to 2023, 1,285 articles in related fields were published worldwide, and the number of articles is on the rise (Figure 2A). The number of articles published in 2013 and 2016 was highlighted, suggesting that attention to CKD and lipid metabolism has changed over time. The number of papers published annually reflects the development of this research field. Among the top ten countries in the number of publications, the number of publications in the United States continues to occupy an important position, with earlier research timelines. Conversely, the proportion of publications in China has gradually increased since 2017 (Figure 2B). Among these, 14 countries published more than 25 articles during the 20 years (Figure 2C).

### 3.2 Publications of major countries worldwide

**FIGURE 3:**
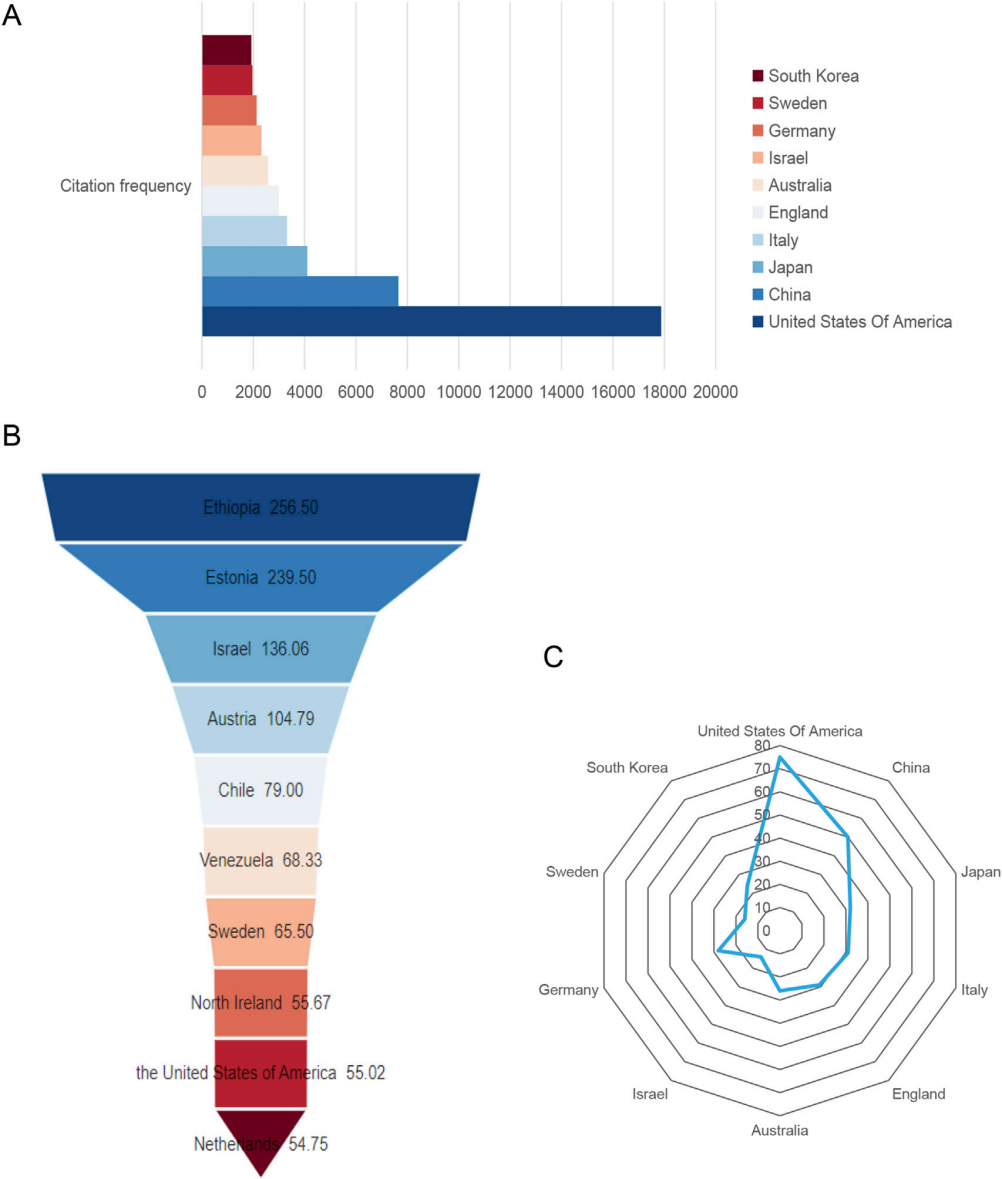
Global citation of relevant publications to assess the quality of publications in individual countries. **(A)** Top ten most frequently cited countries; **(B)** Top ten countries with the highest average citation frequency; **(C)** H-index of the top ten most frequently cited countries.

Figure 3A and Table 1 show that publications in the United States have the most citations (17,880 times), followed by China (7,650 times) and Japan (4,101 times). Israel had the highest average citation frequency (136.06 times). Figure 3B shows that Ethiopia had the highest average citation frequency (256.50 times), and the United States ranks 9th. As shown in Figure 3C and Table 1, among the top ten countries with citations, the H-index of the United States was the highest (75), followed by China (50).

**TABLE 1:** Top ten most frequently cited countries.

Country/Area	Citation frequency	Average citations	H-index
United States of America	17,880	55.02	75
China	7,650	20.73	50
Japan	4,101	37.97	32
Italy	3,312	46.00	31
England	2,976	54.11	29
Australia	2,571	52.47	26
Israel	2,313	136.06	14
Germany	2,129	32.26	28
Sweden	1,965	65.50	16
South Korea	1,922	49.28	24

### 3.3 Publication quality of global core authors

**FIGURE 4:**
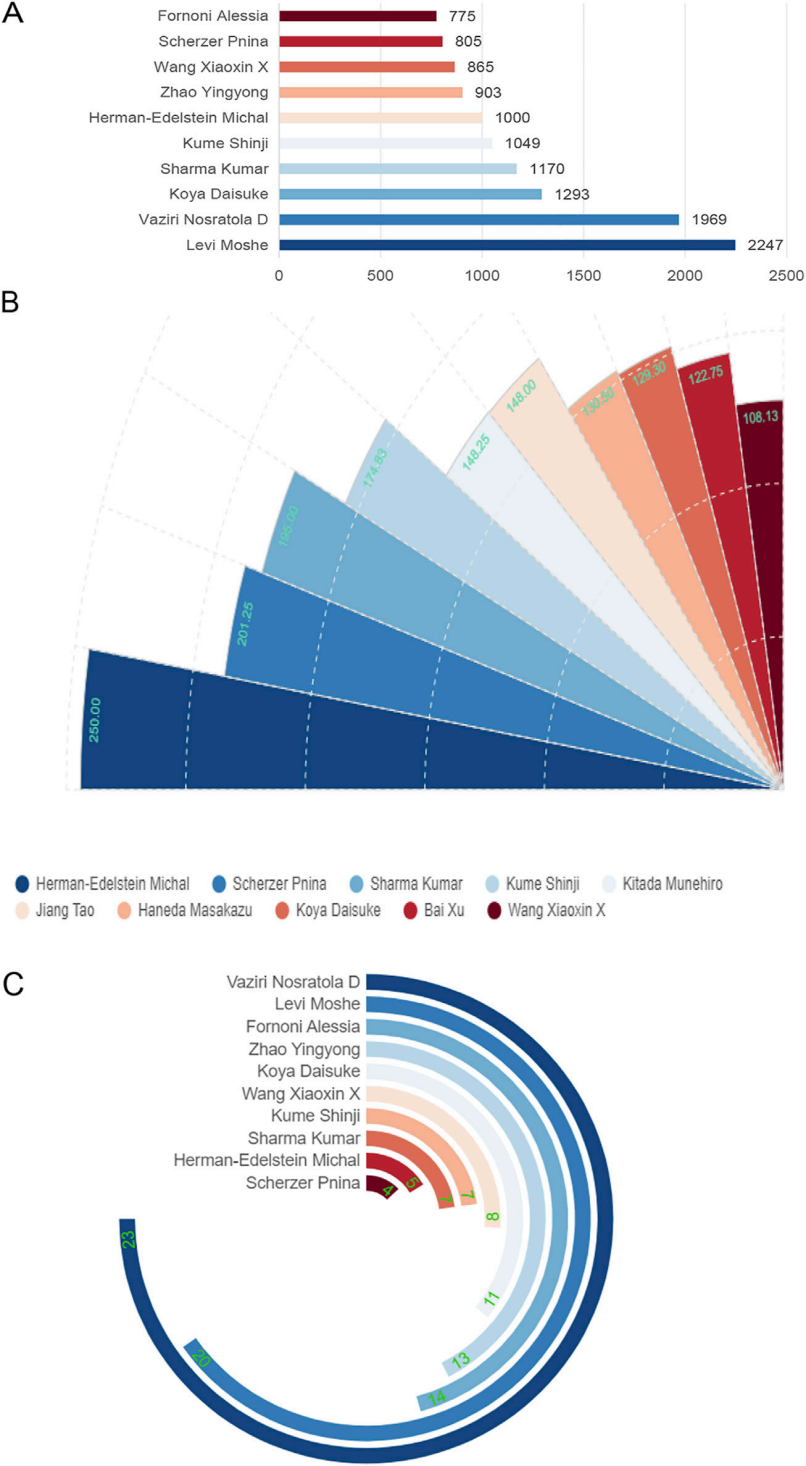
Global citation of relevant publications to assess the quality of individual authors’ publications. **(A)** Top ten most frequently cited authors; **(B)** Top ten authors with the highest average citation frequency; **(C)** H-index of the top ten most frequently cited authors.

The citation frequency of publications reflects the value of the authors. Figure 4A and Table 2 show that the author whose publications were cited most frequently was Levi Moshe (2,247 times), followed by Vaziri Nosratola D (1969 times). Figure 4B shows that Herman-Edelstein Michal publications were cited first on average (250 times), followed by Scherzer Pnina (201.25 times). While Herman-Edelstein Michal has an H-index of only 5, Vaziri Nosratola D has the highest H-index (23) and most published article (25).

**TABLE 2:** Top ten most frequently cited authors.

Author	Publications	Citation frequency	Average citations	H-index
Levi Moshe	21	2,247	107.00	20
Vaziri Nosratola D	25	1,969	78.76	23
Koya Daisuke	10	1,293	129.30	11
Sharma Kumar	6	1,170	195.00	7
Kume Shinji	6	1,049	174.83	7
Herman-Edelstein Michal	4	1,000	250.00	5
Zhao Yingyong	12	903	75.25	13
Wang Xiaoxin X	8	865	108.13	8
Scherzer Pnina	4	805	201.25	4
Fornoni Alessia	18	775	43.06	14

### 3.4 Countries, institutions, authors, and journals published in the field of research

**FIGURE 5:**
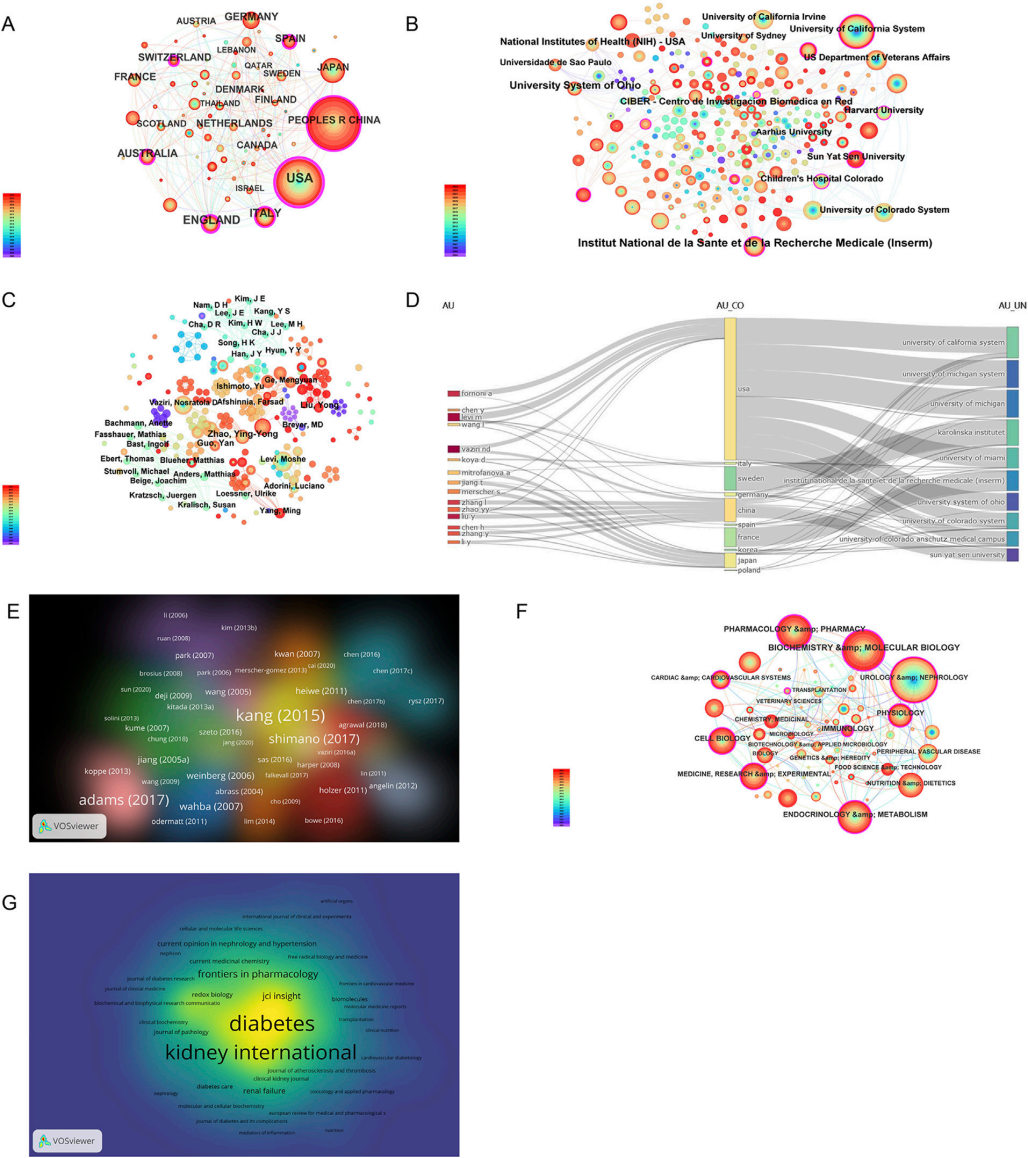
Visual analysis of global publications by country, institution, author, highly cited articles, disciplines, and journals. **(A)** Country visualization diagram; **(B)** Institution visualization diagram; **(C)** Author visualization diagram; **(D)** Relationships among authors, countries and institutions; **(E)** Publication visualization diagram, with label size indicating citation frequency; **(F)** Discipline visualization diagram; **(G)** Journal visualization diagram, with label size representing total link strength. Among **(A, B, C, F)**, the node size represents the number of published papers, and the node label size represents the degree value.

The number of publications also reflects the popularity of research in related fields. Sixty-nine countries and regions have contributed to research on CKD and lipid metabolism. As shown in Figure 5A; Table 3, China had the largest number of publications (368), followed by the United States (325). The number of publications from China and the United States was more than half of the total number of publications. A total of 1,885 institutions were involved in publishing articles in this field. As shown in Figure 5B, the University of California System, US Department of Veterans Affairs, Veterans Health Administration (VHA), University of California Irvine, and University of Colorado System were the top five institutions with the most publications. A total of 7,615 authors have published articles in this field. As shown in Figure 5C, Vaziri Nosratola D, Levi Moshe, Fornoni Alessia, Zhao Yingyong, and Merscher Sandra are the core authors of this field. Figure 5D shows that the core authors and institutions were primarily concentrated in China and the United States. A total of 466 journals have been published in this field. Table 4 shows that the International Journal of Molecular Sciences has the largest number of publications (35 articles). It is a biological journal, and its articles are primarily collected from the fields of biochemical, molecular biology, and chemical synthesis. Owing to the expansion of journals, the number of articles published has risen rapidly since 2019. Kidney International was the most frequently cited journal (2,056 articles). It is an international journal featuring comprehensive research in medicine, urology, and nephrology. It focuses on key research and frontier progress in these fields, making it one of the most frequently cited journals in nephrology.

**TABLE 3:** Top ten countries with the highest number of publications.

Country/Area	Publications	Degree	Centrality
China	368	16	0.19
United States of America	325	35	0.49
Japan	108	14	0.08
Italy	72	21	0.14
Germany	66	20	0.1
Poland	57	4	0
England	55	26	0.24
Australia	49	20	0.16
Spain	41	16	0.1
South Korea	39	5	0

**TABLE 4:** Top ten journals with the largest number of publications.

Journal	Publication numbers	Citation frequency	Average citations	H-index	IF (2022)
International Journal of Molecular Sciences	35	974	27.83	16	5.6
PLoS One	32	1,166	36.44	21	3.7
American Journal of Physiology-Renal Physiology	30	1991	66.37	21	4.2
Frontiers in Pharmacology	29	278	9.59	11	5.6
Kidney International	26	2,056	79.08	24	19.6
Nutrients	25	327	13.08	10	5.9
Frontiers in Endocrinology	24	268	11.17	9	5.2
Scientific Reports	21	480	22.86	12	4.6
Renal Failure	19	269	14.16	10	3.0
Nephrology Dialysis Transplantation	18	648	36.00	14	6.1

### 3.5 Visual analysis of highly cited articles, disciplines, and journals in global publications

As shown in Figure 5E and Table 5, high-quality articles were obtained by analyzing publications in the research field. Among these, the article published by Kang Hyun Mi in Nature Medicine in 2015 was cited the most frequently (869 times). In this thesis, a genome-wide transcriptome study of a large cohort (n = 95) of normal and fibrotic human tubular samples was performed, followed by systems and network analyses, to identify inflammation and metabolism as the most important dysregulated pathways in diseased kidneys. Additionally, both *in vitro* and mouse models of renal tubulointerstitial fibers suggest that correcting defects in fatty acid oxidation (FAO) metabolism might be helpful for the prevention and treatment of CKD (Kang et al., 2015). As shown in Figure 5F, urology and nephrology, biochemistry and molecular biology, endocrinology and metabolism, pharmacology and pharmacy, and medicine, research, and experimental are the key related disciplines in this field. In Figure 5G, Diabetes and Kidney International were the key journals in this field. Diabetes, founded in 1952, is an international journal featuring comprehensive research on medical endocrinology and metabolism that focuses on key research and frontier progress in these fields.

**TABLE 5:** Top ten cited articles.

Title	Author	Year	Journal name	IF (2022)	Citation frequency	Reference
• Defective fatty acid oxidation in renal tubular epithelial cells has a key role in kidney fibrosis development	Kang Hyun Mi	2015	Nature Medicine	82.9	869	Kang et al. (2015)
• Non-alcoholic fatty liver disease and its relationship with cardiovascular disease and other extrahepatic diseases	Adams Leon A	2017	Gut	24.5	697	Adams et al. (2017)
• SREBP-regulated lipid metabolism: convergent physiology - divergent pathophysiology	Shimano Hitoshi	2017	Nature Reviews Endocrinology	40.5	582	Shimano and Sato (2017)
• Obesity-related glomerulopathy: clinical and pathologic characteristics and pathogenesis	D’Agati Vivette D	2016	Nature Reviews Nephrology	41.5	396	D’Agati et al. (2016)
• Obesity and obesity-initiated metabolic syndrome: mechanistic links to chronic kidney disease	Wahba Ihab M	2007	Clinical Journal of the American Society of Nephrology	9.8	381	Wahba and Mak (2007)
• Dyslipidemia of chronic renal failure: the nature, mechanisms, and potential consequences	Vaziri Nosratola D	2006	American Journal of Physiology-Renal Physiology	4.2	360	Vaziri (2006)
• Altered renal lipid metabolism and renal lipid accumulation in human diabetic nephropathy	Herman-Edelstein Michal	2014	Journal of Lipid Research	6.5	358	Herman-Edelstein et al. (2014)
• Mitochondrial dysfunction in diabetic kidney disease	Forbes Josephine M	2018	Nature Reviews Nephrology	41.5	300	Forbes and Thorburn (2018)
• Lipotoxicity	Weinberg JM	2006	Kidney International	19.6	294	Weinberg (2006)
• Exercise training for adults with chronic kidney disease	Heiwe Susanne	2011	Cochrane Database of Systematic Reviews	8.4	290	Heiwe and Jacobson (2011)

### 3.6 Bibliometric analysis of reference, journal, and author citation bursts

**FIGURE 6:**
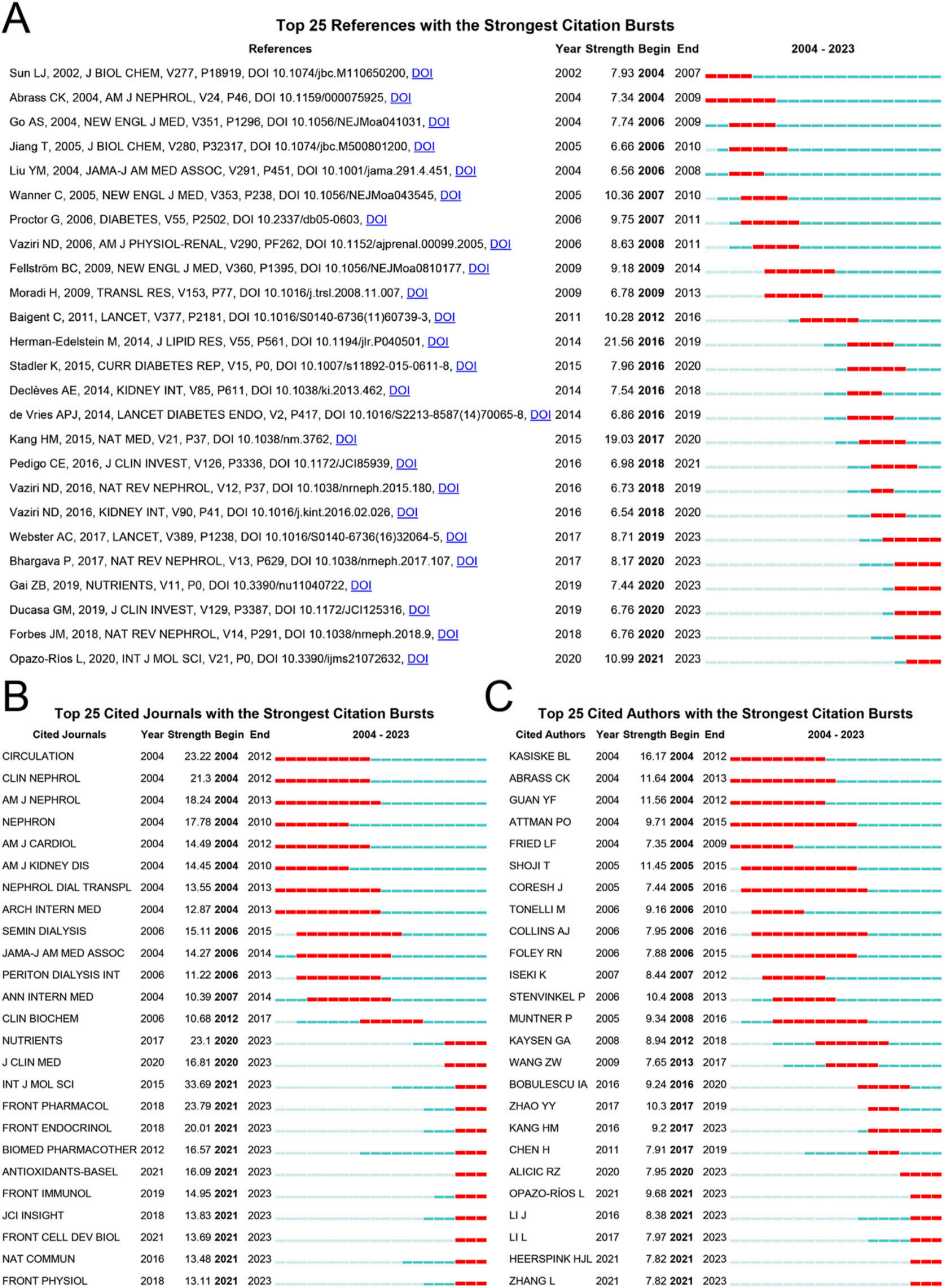
Bibliometric analysis of citation burst among references, journals, and authors. **(A)** Citation burst in references; **(B)** Citation burst in journals; **(C)** Citation burst in authors. Red horizontal lines indicate the importance of references, journals, and authors in the field. The longer the red line, the longer the reference, the journal, and the author sustained attention.

A citation burst is the focus of researchers over a short period, reflecting whether the author’s research is close to the current hotspot. As shown in Figure 6A, the article Altered renal lipid metabolism and accumulation in human diabetic nephropathy (DN) published by Herman-Edelstein Michal in 2014, showed the highest burst intensity (21.56) with 358 citations. This study examined lipid staining and lipid metabolism gene expression in renal biopsies from patients with DN (n = 34) and compared them with those in normal kidneys (n = 12). Massive lipid deposition and increased intracellular lipid droplets were associated with dysregulated lipid metabolism genes, and there was a highly significant correlation among glomerular filtration rate, inflammation, and lipid metabolism genes. This provides evidence for the pathogenic mechanism of lipid metabolism in DN (Herman-Edelstein et al., 2014). Rosuvastatin and cardiovascular events in patients undergoing hemodialysis, published by Fellström in 2009 in the New England Journal of Medicine, showed the most sustained burst intensity (9.18, 2009–2014). A multicenter, randomized, double-blind trial enrolled 2,776 patients to study the improvement of cardiovascular events in maintenance hemodialysis patients treated with rosuvastatin. The trial demonstrated that rosuvastatin reduced low-density lipoprotein cholesterol levels but did not have a significant effect on the composite primary endpoints of death from cardiovascular causes, nonfatal myocardial infarction, or nonfatal stroke (Fellstrom et al., 2009). An analysis of the journals shows in Figure 6B that the International Journal of Molecular Sciences, which focuses on chemistry, molecular physics (chemical physics and physical chemistry), and molecular biology, has the strongest burst intensity (33.69). Various journals have been published for a long time. In Figure 6C, Attman PO and Coresh Josef are the authors with the longest duration of burst intensity, both spanning 11 years, with outbreak intensities of 9.71 and 7.44, respectively. Among them, Attman PO has published articles in Kidney International (five articles), American Journal of Kidney Diseases (two articles), Journal of the American Society of Nephrology (one article), and Progress in Lipid Research (one article) from 1987 to 2006. Additionally, Coresh Josef has published several articles in the field of CKD and lipid metabolism (Attman et al., 1987; Attman and Alaupovic, 1991; Attman et al., 1992; Attman et al., 1993; Samuelsson et al., 1998; Attman et al., 1999; Lee et al., 2002; Samuelsson et al., 2002; Alaupovic et al., 2006). Josef published several articles in various top journals focusing on cardiac cardiovascular systems and urology and nephrology (Buckley et al., 2023; Deo et al., 2023; Khan et al., 2023; Ndumele et al., 2023a; Ndumele et al., 2023b). Kasiske Bertram L is the author with the strongest burst intensity (16.17) and has published several articles in a variety of top journals; however, the main area of research is Transplantation (Lam et al., 2015; Grams et al., 2016; Wheeler and Kasiske, 2016; House et al., 2019; Kasiske et al., 2020; Josephson et al., 2023).

### 3.7 Collaborative analysis between authors, countries, and institutions

**FIGURE 7:**
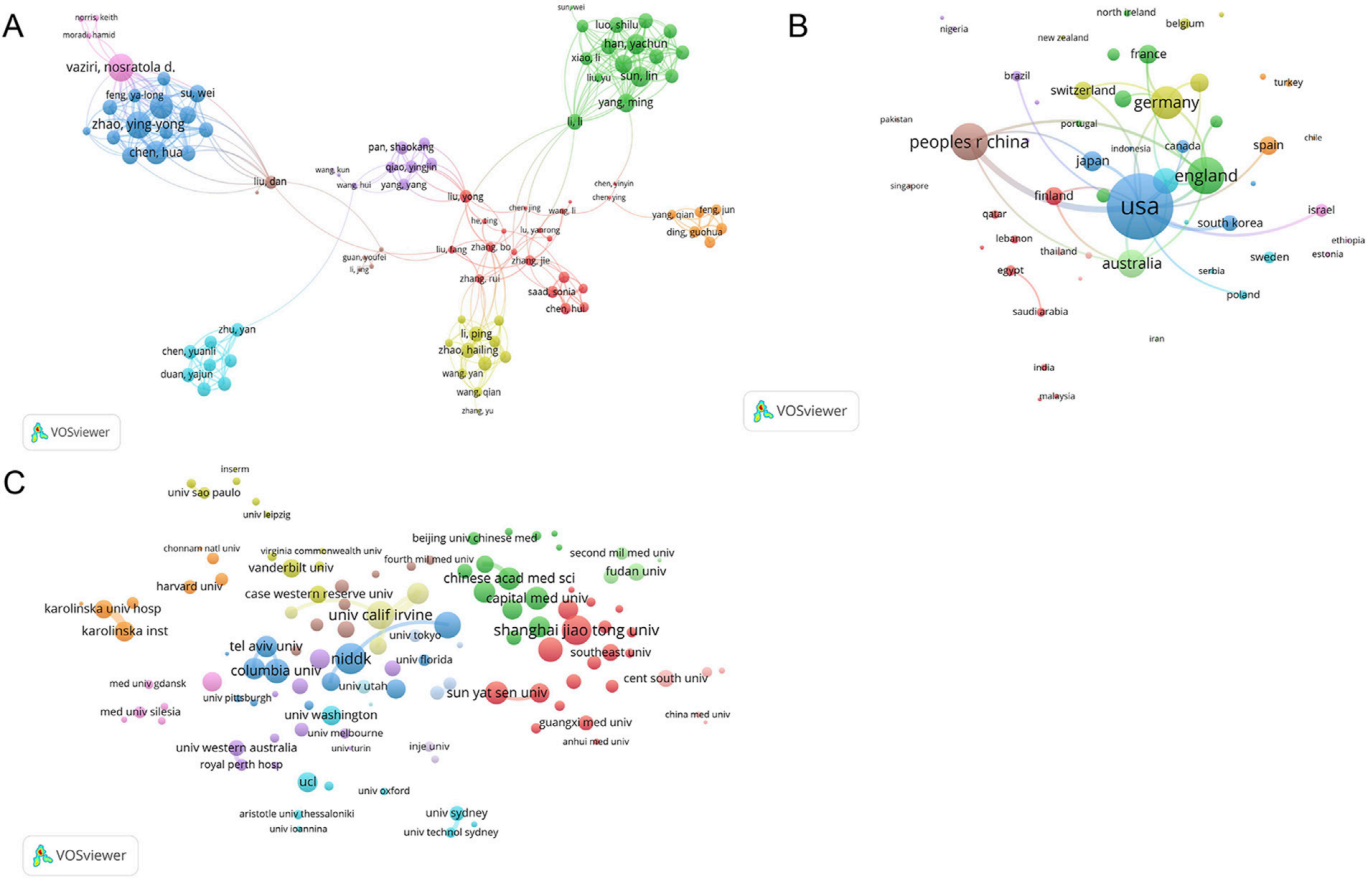
Map of collaboration among authors, countries, and institutions. **(A)** Visualization of authors co-cited by publications; **(B)** Visualization of countries of cooperation between publications; **(C)** Visualization of cooperative institutions among publications. Different colors represent different clusters. Nodes of the same color represent authors, countries, and institutions, showing closer and more frequent collaboration. The size of each node represents the total link strength. The lines between nodes indicate cooperation, and the width of the lines indicates the degree of cooperation.

Co-citation occurs when an article is co-cited by different authors or when an author is cited multiple times. Co-citations reflect the research direction and cross-developments of academia. Figure 7A shows the cooperative circle of authors formed in this field. Vaziri Nosratola D and Zhao Yingyong collaborated closely and jointly on several publications (Chen et al., 2019; Feng et al., 2020). Figure 7B shows that the United States, England, and China are the core contributing countries. The National Institute of Diabetes and Digestive and Kidney Diseases (NIDDK), Shanghai Jiaotong University, and University of California, Irvine, were the core contributing institutions (Figure 7C).

### 3.8 Analysis of co-cited publications and journals

**FIGURE 8:**
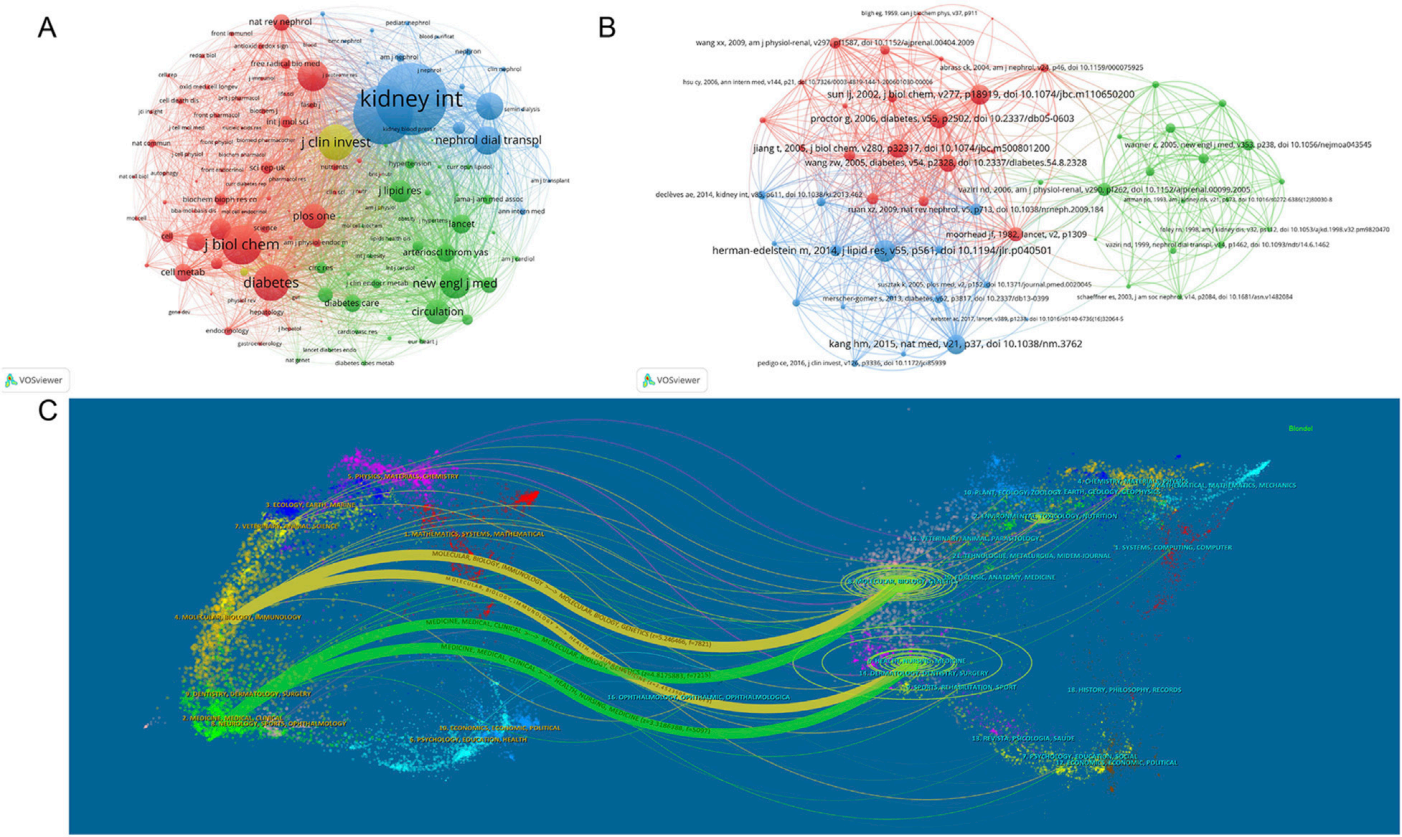
Visual analysis of co-cited publications and journals. **(A)** Visualization of collaborative journals between co-cited publications; **(B)** Visualization of co-cited publications; **(C)** Overlay analysis of journal double graphs. Different colors in A and B represent different clusters. Nodes of the same color represent journals and publications exhibiting more frequent citations. The size of each node represents the total link strength. The lines between nodes indicate reference relationships, and the width of the lines indicates the degree of reference. In C, the left side is the citing journal, the right side is the cited journal, and the line path represents the citation relationship.

The cocitation map reveals the cocitation network of highly cited articles. As shown in Figure 8A, Kidney International and the Journal of the American Society of Nephrology were the core contributing journals. In Figure 8B, Herman-Edelstein Michal’s article, published in the Journal of Lipid Research in 2014, was the core and most co-cited document (133 times). As shown in Figure 8C, the published articles were primarily in the fields of molecular biology, immunology, medicine, medical and clinical, whereas the cited articles were mainly in the fields of molecular biology, genetics, health, nursing, and medicine.

### 3.9 Keyword analysis and future direction

**FIGURE 9:**
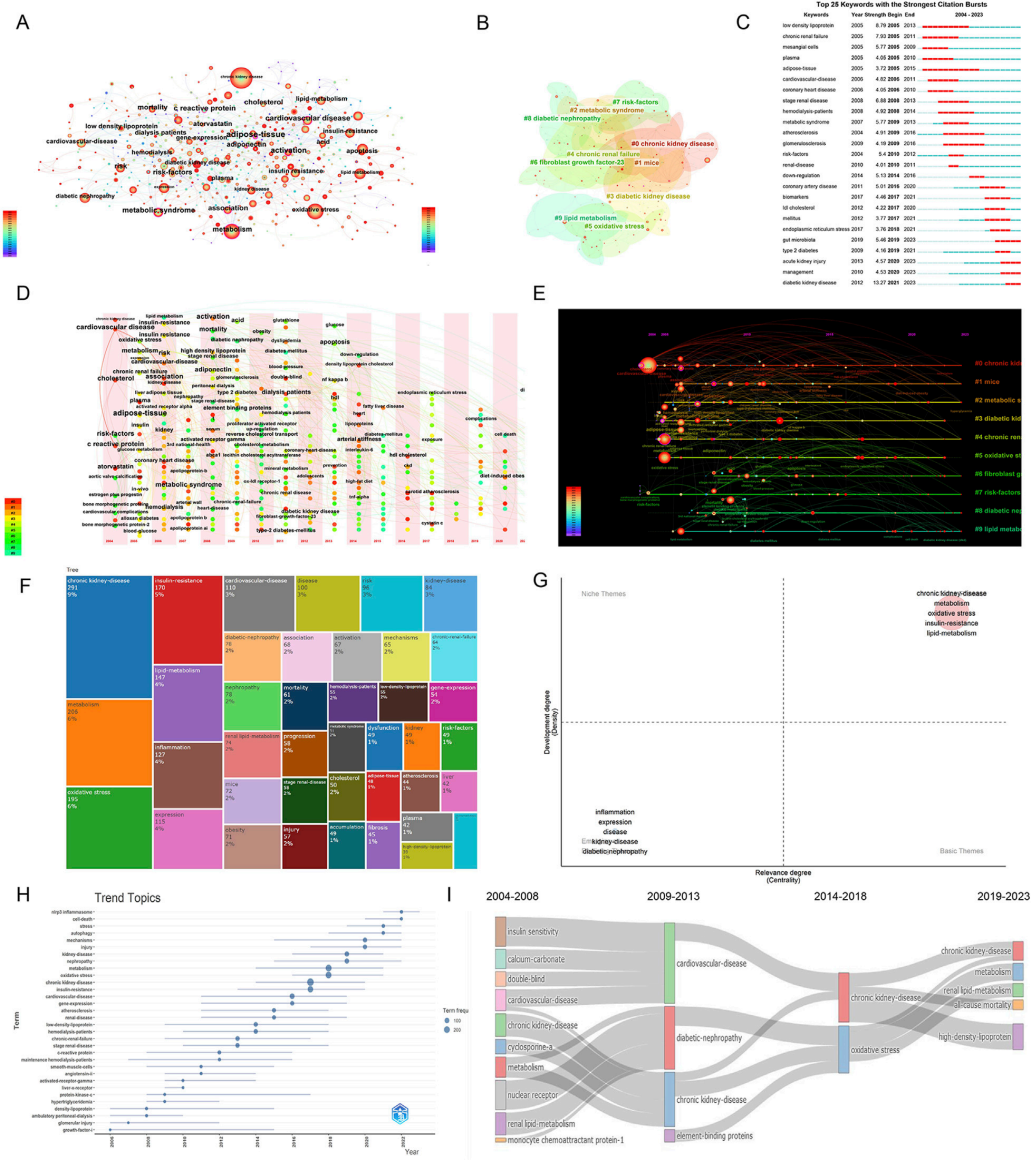
Visual analysis of keywords and trends in research hotspots. **(A)** Keyword visualization diagram, where node size represents the occurrence frequency and node label size represents the degree value; **(B)** Keywords network cluster analysis, clusters with different colors represent different clusters; **(C)** Keyword burst analysis; **(D)** Keywords time zone map; **(E)** Keyword time map; **(F)** Tree map; **(G)** Thematic map; **(H)**. Trend topics; **(I)** Thematic evolution.

Keyword analysis can identify field hotspots and potential research directions. Figure 9A and Table 6 show high-frequency keywords: chronic kidney disease (436 times), oxidative stress (238 times), metabolism (205 times), inflammation (125 times), and lipid metabolism (117 times). The visible keywords in Figure 9B can form ten clusters, which indicate the most prominent themes in the field to date. As shown in Figure 9C, adipose tissue was the most persistent keyword, as the research heat persisted for 10 years. Diabetic kidney disease (DKD) had the highest outbreak intensity (13.27). Further analysis of the clusters (Figures 9D, E) showed that cardiovascular disease (CVD) and CKD have been popular research topics since 2004, indicating a close relationship between the two. DKD has also gradually become a popular research topic in this field.

**TABLE 6:** High frequency keywords related to lipid metabolism in chronic kidney disease.

Keyword	Frequency	Degree	Centrality
Chronic kidney disease	436	31	0.05
Oxidative stress	238	41	0.09
Metabolism	205	42	0.07
Inflammation	125	23	0.02
Lipid metabolism	117	29	0.05
Expression	115	32	0.05
Diabetic nephropathy	113	27	0.03
Insulin-resistance	99	35	0.07
Disease	98	40	0.07

As shown in Figures 9F–I, CKD and abnormal lipid metabolism have been the focus of research, among which oxidative stress (6%), inflammation (4%), and insulin resistance (IR) (5%) are crucial. Figures 9H, I show the changing trend of keywords, among which autophagy, the NOD-like receptor protein 3 (NLRP3) inflammasome, and cell death have been the research hotspots in recent years.

## 4 Discussion

From 2004 to 2023, the number of publications on lipid metabolism in CKD gradually increased, indicating that lipid metabolism in CKD will remain a research hotspot in the future. In this study, we provide a comprehensive overview of the progress in lipid metabolism research in CKD through bibliometric analysis, with a focus on future research directions. Globally, the authors, countries, and institutions in this field are primarily concentrated in China and the United States. Vaziri Nosratola D, Levi Moshe, and Zhao Yingyong were the main contributing authors, who were the authors of the top five publications and top ten cited publications. There is also a close collaboration between Vaziri Nosratola D and Zhao Yingyong. The identification of serum metabolites associated with chronic kidney disease progression and the antifibrotic effect of 5-methoxytryptophan (5-MTP) was published in Nature Communications in 2019 and is one of the most cited papers in their collaboration (165 times) (Chen et al., 2019). In this study, the level of 5-MTP decreased with the progression of CKD according to untargeted metabolomics in 2,155 patients with CKD and healthy controls. The efficacy of 5-MTP was further verified in mice with unilateral ureteral obstruction, ischemia/reperfusion injury, and in human kidney cells. The key role of tryptophan hydroxylase-1, an enzyme involved in 5-MTP synthesis, in inhibiting renal inflammation and fibrosis has been confirmed. They also showed that serum acetylcarnitine levels in patients with CKD increased significantly with a decrease in renal function. Acetylcarnitine may facilitate acetyl-CoA uptake into the mitochondrial matrix during FAO. Elevated serum acetylcarnitine levels in patients with CKD can contribute to skeletal muscle IR by promoting the reverse carnitine acetyltransferase reaction, leading to the accumulation of acetyl-CoA in the mitochondria (Miyamoto et al., 2016). In a study using UPLC-HDMS-based lipidomics to detect serum lipid metabolites in 180 patients with advanced CKD and 120 healthy controls, elevated levels of serum total fatty acids, glycerides, and glycerophospholipids were directly correlated with elevated serum triglyceride levels and inversely correlated with estimated glomerular filtration rate (eGFR) levels. Changes in lipid metabolites have also been reported in patients with CKD (Chen et al., 2017). Polyporus umbellatus may play a protective role in renal fibrosis by regulating fatty acyl metabolism (Wang et al., 2021).

Kidney International, a leading journal in the field of kidney disease, has published several articles on lipid metabolism in patients with CKD. The article on Lipotoxicity published by Weinberg JM in the Journal in 2006 was also among the top ten most-cited articles in the field (294 times) (Weinberg, 2006). This review summarizes the related findings on non-renal tissue lipotoxicity and the pathogenesis of renal lipotoxicity in acute and CKD to further promote the development of this field.

The keywords summarize developments in the field. Systemic lipid homeostasis is of great interest in cardiometabolic diseases (Arsenault et al., 2011; Jaffe and Karumanchi, 2024; Maestri et al., 2024). Glucose and lipid metabolism disorders are the major determinants of the development and progression of DKD (Fu et al., 2020; Mori et al., 2021). Our keyword analysis showed that CVD and DKD are hotspots in the study of lipid metabolism in CKD. Dyslipidemia is a major risk factor for CVD (Hager et al., 2017). Elevated triglyceride levels may increase the risk of CVD through excessive free fatty acid release, the production of proinflammatory cytokines and coagulation factors, and fibrinolysis dysfunction (Reiner, 2017). Lipid oxidation promotes atherosclerosis through endothelial dysfunction and inflammation (Gianazza et al., 2019). CKD is a major risk factor for atherosclerotic CVD (Tonelli et al., 2012). Patients with CKD were also associated with a significant cardiovascular risk (OR = 1.69), and approximately 50% of patients with CKD4-5 had cardiovascular disease (Stevens et al., 2007). Its manifestations include coronary artery disease, heart failure, arrhythmias, and sudden cardiac death (Jankowski et al., 2021). CVD was the most common cause of death in patients with CKD3-5. With a decrease in eGFR, the proportion of deaths from heart failure and valvular disease increased significantly (Thompson et al., 2015). Increasing evidence suggests that lipid-lowering therapy can reduce the incidence of cardiovascular events in CKD (Schlackow et al., 2019). DKD is the leading cause of ESRD (Umanath and Lewis, 2018). Abnormal glucose and lipid metabolisms play important roles in the pathogenesis of DKD. In DKD, ectopic lipid accumulation in the kidney exceeds lipid droplet storage and damages podocytes and tubular cells (Yang et al., 2018; Schelling, 2022). Studies have shown that Statins are effective in preventing DKD (Zhou et al., 2023). Therefore, correcting dyslipidemia has great potential to delay the progression of CKD.

According to the keyword trends, inflammation, oxidative stress, and IR were the topics of lipid metabolism. Inflammation is the main pathogenic mechanism underlying CKD, obesity, impaired glucose tolerance, and IR (Brennan et al., 2021), which can lead to glomerulosclerosis, tubular atrophy, and fibrosis. Ectopic lipid deposition in the kidneys can lead to an inflammatory response (Samuel and Shulman, 2012). Studies have shown that DKD is associated with proinflammatory cytokines, such as circulating levels of C-reactive protein, interleukin-6, intercellular adhesion molecule-1, plasminogen activator inhibitor 1, soluble tumor necrosis factor receptor-1 (sTNFR-1), and sTNFR-2 (Groop et al., 2009; Lopes-Virella et al., 2013; Liu et al., 2024). NLRP3 is a prominent target in the study of inflammatory mechanisms. ZDHHC12-mediated palmitoylation promotes NLRP3 degradation via the chaperone-mediated autophagy pathway (Wang and Cui, 2023). Fatty acid synthesis can drive inflammation by activating the NLRP3 inflammasome through palmitoylation (Leishman et al., 2024). In addition, statins can inhibit NLRP3 activation in the kidney to improve cholesterol-induced inflammation and water channel aquaporin 2 (AQP2) expression (Kong et al., 2020). 6-Gingerol plays a crucial protective role in DN by inducing the expression of miRNA-146a and miRNA-223 and inhibiting the TLR4/TRAF6/NLRP3 inflammasome signaling pathway (Aboismaiel et al., 2024). Oxidative stress is also associated with CKD (Kishi et al., 2024). Lipotoxicity is a significant inducer of oxidative stress (Tanase et al., 2022; Chae et al., 2023). Lipid peroxidation can regulate podocyte migration and cytoskeletal structure through redox-sensitive Ras homologous gene family member A signaling (Kruger et al., 2018). The cellular oxidative stress microenvironment can drive fibroblast activation, leading to renal fibrosis (Li et al., 2023). Research on intracellular signal transduction pathways induced by oxidative stress focuses on the kelch-like erythroid cell-derived protein with the CNC homology-associated protein 1(Keap1)- nuclear factor erythroid 2-related factor 2 (Nrf2) pathway, forkhead box O (FoxO) proteins, HIF-1 pathway and the nuclear factor-κB (NF-κB) pathway (Chae et al., 2023). A meta-analysis of 95 studies suggested that antioxidants may reduce cardiovascular events and progression to renal failure and may improve renal function (Colombijn et al., 2023). Increasing trends in IR were associated with a high risk of adverse renal outcomes, and eGFR was independently associated with insulin sensitivity (Akwo et al., 2021; Yang et al., 2022). The exacerbation of IR increases the risk of cardiorenal outcomes in CKD, whereas the endothelin receptor antagonist atrisentan reduces IR (Smeijer et al., 2023). Farnesoid X receptor agonists also improve IR and renal lipid metabolism in db/db mice (Han S. Y. et al., 2021).

Recently, autophagy and cell death have attracted considerable attention in the field of lipid metabolism in CKD. Autophagy affects different renal cell types and significantly affects the maintenance of renal function and homeostasis (He et al., 2014). It may exert a protective effect against cyclosporin-induced metabolic stress in renal proximal tubular epithelial cells (Kimura et al., 2013). Autophagy in endothelial cells and podocytes synergistically protects against diabetes-induced glomerulosclerosis (Lenoir et al., 2015). Lipophagy is selective autophagy that targets lipid droplets for degradation (Laval and Ouimet, 2023). In proximal tubule cell (PTC)-specific Atg5-deficient (atg5-TSKO) mice, lipophagy counteracts prolonged starvation in PTCs to prevent cellular energy depletion (Minami et al., 2017). Lipophagy is involved in ectopic lipid deposition and lipid-related renal injury in DN; however, these changes can be reversed by AdipoRon, an activator of the adiponectin receptor that promotes autophagy (Han Y. et al., 2021). Deficiency of α-klotho, an anti-aging protein in the renal tubular epithelial cell membrane, leads to ubiquitin-mediated adipose triglyceride lipase degradation, which is a common molecular basis for lipolysis and lipophagy (Wang et al., 2023). Autophagy-modifying drugs can be used to prevent or treat kidney disease. Metformin can upregulate adenosine monophosphate-activated protein kinase signaling and induce autophagy (Lin et al., 2019). Autophagy inhibition can activate endoplasmic reticulum stress, leading to podocyte apoptosis (Fang et al., 2014). Cell death is critical in the pathophysiology of CKD. Cholesterol 25-hydroxylase, an enzyme involved in cholesterol metabolism, maintains endothelial cell activity by regulating ADP-ribosylation factor 4, which further improves kidney injury in DKD (Zhang et al., 2024). Necroptosis leads to the release of cellular contents and cytokines that trigger an inflammatory response in adjacent tissues (Kolbrink et al., 2023). TNF-α and interferon-γ can induce necroptosis through two different pathways (Liu et al., 2018). Ferroptosis is a recently discovered form of iron-dependent cell death characterized by the accumulation of lipid peroxides (Li S. et al., 2024). It depends on the convergence of iron, thiol, and lipid metabolic pathways (Bayır et al., 2023). Ferroptosis plays a crucial role in renal fibrosis, which is a key pathological change in CKD (Li S. et al., 2024). The ferroptosis inducers, erastin and RSL3, can induce renal tubular epithelial cell death *in vitro* (Wang et al., 2020). Ferroptosis inhibitor Ferrostatin-1 (Fer-1) alleviates renal tubular cell death induced by transforming growth factor-beta1 (Kim et al., 2021). In summary, numerous studies exist on the mechanism of lipid metabolism in CKD; however, effective treatment of CKD with related targets must be further studied.

We found that lipid metabolism in IgA nephropathy, membranous nephropathy, and other types of glomerulonephritis has been under-researched. There is significant potential for research on drugs that target renal lipid metabolism therapy (Izumihara et al., 2024). The discovery of peptide drugs for the treatment of CKD has attracted wide attention, with fibroin peptide notably ameliorating adriamycin-induced nephropathy by regulating lipid metabolism through the PANX1-PPARα/PANK1 pathway (Li S. S. et al., 2024). Our study also highlighted traditional Chinese medicine monomers as a promising area for maintaining renal lipid homeostasis, such as hydrangea paniculata coumarins (Chen et al., 2024), Icariside II (Wang et al., 2024), berberine (Qin et al., 2020), etc.

Our study has some limitations. First, the data for this study were only obtained from the WOSCC database, and some articles in non-English, non-research, and reviews were not included. Second, the data in this study were obtained using bibliometric software based on machine learning, and most of the results were obtained using machine algorithms; therefore, some new research ideas may not be presented. Third, the latest high-quality articles could not be included in the statistics, possibly because of insufficient citation frequency due to time constraints.

## 5 Conclusion

In this study, we conducted a systematic bibliometric review of CKD and lipid metabolism from 2004 to 2023 to demonstrate the current status and global trends in related fields. China has the most publications and is second only to the United States in H-index and number of citations in this field. The International Journal of Molecular Sciences published the most publications in this field, and Kidney International, the top journal in the field of kidney disease, is also a core publication. CVD and DKD are closely associated with the lipid metabolism of patients with CKD. Additionally, oxidative stress, inflammation, IR, autophagy, and cell death are research hotspots. Future studies should focus on the treatment of CKD with lipid-lowering targets.

## Data Availability

The original contributions presented in the study are included in the article, further inquiries can be directed to the corresponding authors.
